# A few strong connections: optimizing information retention in neuronal avalanches

**DOI:** 10.1186/1471-2202-11-3

**Published:** 2010-01-06

**Authors:** Wei Chen, Jon P Hobbs, Aonan Tang, John M Beggs

**Affiliations:** 1 Indiana University Department of Physics, 727 East 3rd Street, Bloomington, Indiana, USA

## Abstract

**Background:**

How living neural networks retain information is still incompletely understood. Two prominent ideas on this topic have developed in parallel, but have remained somewhat unconnected. The first of these, the "synaptic hypothesis," holds that information can be retained in synaptic connection strengths, or weights, between neurons. Recent work inspired by statistical mechanics has suggested that networks will retain the most information when their weights are distributed in a skewed manner, with many weak weights and only a few strong ones. The second of these ideas is that information can be represented by stable activity patterns. Multineuron recordings have shown that sequences of neural activity distributed over many neurons are repeated above chance levels when animals perform well-learned tasks. Although these two ideas are compelling, no one to our knowledge has yet linked the predicted optimum distribution of weights to stable activity patterns actually observed in living neural networks.

**Results:**

Here, we explore this link by comparing stable activity patterns from cortical slice networks recorded with multielectrode arrays to stable patterns produced by a model with a tunable weight distribution. This model was previously shown to capture central features of the dynamics in these slice networks, including neuronal avalanche cascades. We find that when the model weight distribution is appropriately skewed, it correctly matches the distribution of repeating patterns observed in the data. In addition, this same distribution of weights maximizes the capacity of the network model to retain stable activity patterns. Thus, the distribution that best fits the data is also the distribution that maximizes the number of stable patterns.

**Conclusions:**

We conclude that local cortical networks are very likely to use a highly skewed weight distribution to optimize information retention, as predicted by theory. Fixed distributions impose constraints on learning, however. The network must have mechanisms for preserving the overall weight distribution while allowing individual connection strengths to change with learning.

## Background

The question of how the brain stores memories has generated intense interest. It is widely thought that information is retained in the strengths of synaptic connections between neurons [1-3]. The "synaptic hypothesis" is supported by numerous studies demonstrating that synaptic strengths do in fact change after learning, and that manipulations that block these changes also interfere with learning [4]. A host of models has explored how such modifiable synapses, when embedded in a network of neurons, could store information [5-8]. In these models, different items are represented by distinct patterns of active neurons in the network. Recent theoretical work has explored how the distribution of connection weights affects the capacity of a network to store such patterns [9-13]. These studies have consistently demonstrated that a skewed distribution, where most weights are relatively weak and only a few are strong, maximizes the number of patterns that can be retained. Electrophysiological studies support this view, and have shown that the distribution of synaptic strengths onto neurons in many parts of the brain is indeed skewed [9,14].

The network activity patterns predicted by the synaptic hypothesis have also found experimental support. Several studies have shown that cortical slice networks, as well as randomly connected networks of cortical neurons, can retain multiple, distinct activity patterns that spontaneously re-occur more often than expected by chance [15-19]. These significantly repeating activity patterns are diverse, temporally precise, and stable for several hours, suggesting that they may be used by the brain to retain information [17,19]. Studies from intact animals are consistent with this, and show that precisely repeating sequences of neural activity occur when birds sing a well-learned song [20-22], or when rats recapitulate a path taken through an often-traveled maze [23-26].

To advance these two lines of work, a link must be made between the distribution of connection strengths and the patterns retained in living neural networks. In our previous work, we reported that isolated cortical networks produced an average of 30 ± 14 (mean ± s.d.) distinct groups of significantly repeating patterns [17]. We later published a model that generated repeating patterns that were remarkably similar to those from experiments [27]. In addition, this model reproduced the size distribution of cascades, called "neuronal avalanches" observed in these networks [27,28]. By tuning the weight distribution in this model and comparing its output to the data from cortical slice networks, we sought to answer two questions: What distribution of connection strengths will best fit the statistics of the observed repeating patterns? What distribution of connection strengths will maximize the number of repeating activity patterns? Our hypothesis was that a skewed distribution would both fit the data and be optimal.

We report here that the distribution of connection strengths that best fits the data is also the distribution that maximizes the number of repeating patterns. This distribution has many weak connections and only a few strong ones. Portions of this work were previously presented as a talk [29] and in abstract form [30].

## Results

### Overview

The results comprise four sections. First, we describe general features of activity from the 60-channel multielectrode array recordings in acute cortical slices. Second, we show that a computational model qualitatively reproduces the avalanche size distribution and the significantly repeating avalanches generated by these slice networks. As very similar findings have been reported previously, these results are presented only briefly for the sake of clarity and completeness. Third, we show that the distribution of connection weights in the model has a large effect on the distribution of repeating activity patterns. Fourth, we show how the distribution of connection weights in the model also affects the overall number of distinct repeating activity patterns.

### Activity produced by acute cortical slices

**Figure 1:**
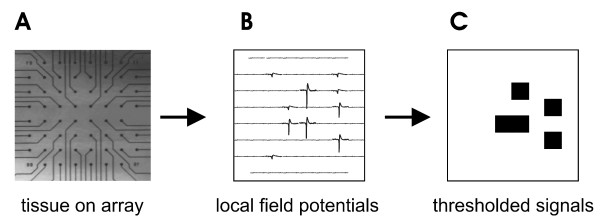
**Electrode array and data representation**. A, Photo of cortical tissue on the 60-channel microelectrode array. Electrodes appear as small black circles at the ends of lines. Interelectrode distance is 200 μm and electrode diameter is 30 μm. B, Arrangement of local field potential (LFP) signals on electrodes. Note that LFPs can vary in amplitude. C, Suprathreshold LFPs represented by small black squares. LFPs that exceed three standard deviations of the mean are considered suprathreshold. All subthreshold LFPs are represented by borderless white squares.

We recorded spontaneous extracellular activity from acute slices of rat somatosensory cortex (n = 7) with 60-channel multielectrode arrays for two hours per preparation. Each slice network had 35 or more active electrodes. Local field potential (LFP) activity from these slices was similar to that reported previously for organotypic cultures [17,28,31] and consisted of quiescent periods punctuated by network bursts. Local field potentials that crossed threshold appeared as negative voltage peaks approximately 20 ms wide, indicative of a population spike (figure 1). Such sharp negative LFPs are thought to be produced by a group of neurons in the vicinity of the electrode firing nearly synchronous action potentials [32]. Data were binned at 4 ms, as this was the average time between successive activation of two electrodes within a network burst when the interelectrode spacing was 200 μm, as reported previously [28].

**Figure 2:**
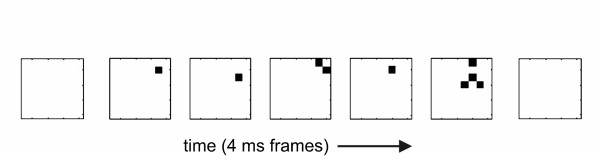
**Example of an avalanche**. Seven frames are shown, where each frame represents activity on the electrode array during one 4 ms time step. Suprathreshold activity at electrodes is shown by small black squares. An avalanche is a series of consecutively active frames that is preceded by and terminated by blank frames. Avalanche length is given by the number of active frames, while avalanche size is given by the total number of active electrodes. The avalanche shown here has a length of 5 and a size of 9.

**Figure 3:**
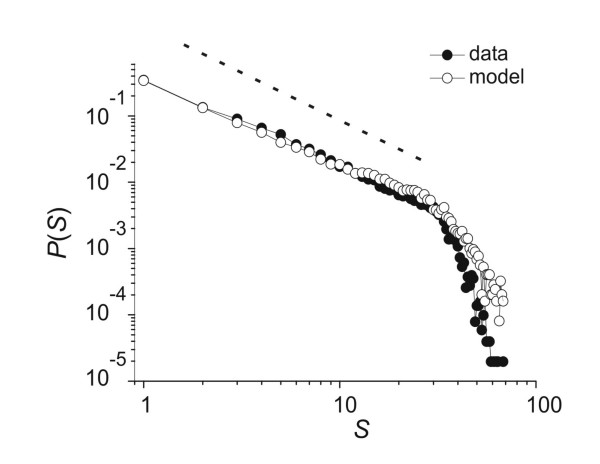
**The avalanche size distribution produced by cortical tissue and by the model**. The probability of observing an avalanche of size *S *is plotted against avalanche size. Distribution from a cortical slice plotted in dark circles shows a linear portion that can be approximated by a power law with slope -3/2, shown as the dashed line. Data deviates from the power law near *S *= 35 because there are only 60 electrodes in the array and many sites are refractory after large events. Distribution from model plotted in open circles closely follows the data.

When activity from all electrodes in an array was plotted in raster form, we commonly observed multiple LFPs in the same time bin. We also observed cascades of consecutively active time bins (figure 2). After cascades were recorded over several hours, it was possible to plot their size distribution (figure 3), which could be approximated by a power law. Because this distribution was not expected by chance [33], but was similar to that produced by computational models of sand pile avalanches [34], we previously named these events "neuronal avalanches" [28].

**Figure 4:**
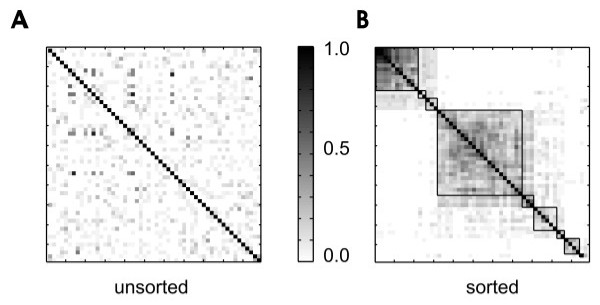
**Sorting a similarity matrix**. A, When *N *avalanches of a given length are compared to each other, their similarity values can be entered in an *N *× *N *matrix as shown here. Each row of the matrix corresponds to one avalanche, and each column within that row indicates how similar that avalanche is to another avalanche. Shaded scale bar ranges from 0 to 1, with greatest similarity coded black. In this figure diagonal elements which compare each avalanche to itself produce perfect similarity. Gray and white pixels appear in the off-diagonal regions, indicating intermediate to low similarity. In this unsorted matrix, the order of avalanches along the margins merely reflects their temporal order of appearance in the recording, and no obvious grouping of dark pixels is found. B, Here avalanches are sorted by an algorithm in order of descending similarity. The highest mutual similarity values appear in the upper left of the matrix, as shown by the darkened pixels. Squares may be drawn to separate dark regions from lighter regions, so as to produce groupings of avalanches.

Another feature of neuronal avalanches reported earlier is that many of them display spatio-temporal structure. When all avalanches of a given length are compared, many of them are more similar to each other than would be expected by chance [17]. We found that spontaneous activity from acute cortical slices shared this property. These repeating avalanches could be ordered into groups (figure 4) that were stable over two hours. This long-term stability, as well as the similarity that these avalanches have to spatio-temporal sequences of activity found in vivo, suggest that neuronal avalanches could be used to retain information in cortical circuits.

### Activity produced by the model

**Figure 5:**
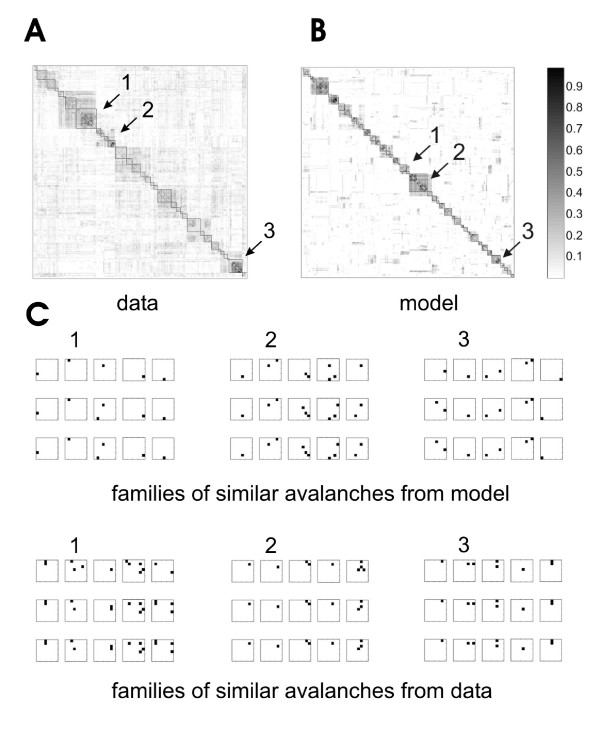
**Repeating avalanches and similarity matrices produced by cortical tissue and by the model**. A, Ordered similarity matrix showing groups of similar avalanches from acute slice data. Matrix of all avalanches of length 5 compared to each other for Boolean similarity. Scale bar indicates similarity (ranging from 0 to 1), with highest similarity coded black. Arrows (*1*: **2**: **3**) point to example regions of the matrix with groups of similar avalanches. Comparable similarity matrices are also found in data from cultures (see [17]. B, Ordered similarity matrix produced by the model appears like matrix from data, with similar sized groups along the diagonal. C, Groups of similar avalanches produced by the model, matching the numbered regions (*1, 2, 3*) from the model's similarity matrix. Note that avalanches within groups share general, not exact, resemblance. D, Groups of similar avalanches produced by the data from regions (*1, 2, 3*) in the data similarity matrix.

We used a simple model based on a branching process [27,35] to capture the central features of the slice data (see Methods). Briefly, each node in the network represented activity at one of the 60 electrodes. When a node became active, it had some probability, *p*, of activating another node in the next time step. In this model, each node had 10 connections to other nodes, and the set of *p *values coming from each node determined the weight distribution. Figure 3 shows that the avalanche size distribution produced by the model is qualitatively similar to that typically produced by an acute cortical slice [27,28]. Figure 5 shows that the repeating avalanches produced by the model are qualitatively similar to those commonly found in the data [17,27]. These results demonstrate that the model is a reasonable platform from which to explore the effects of the weight distribution on significantly repeating avalanches.

### Effect of the exponent *B *on the distribution of groups

**Figure 6:**
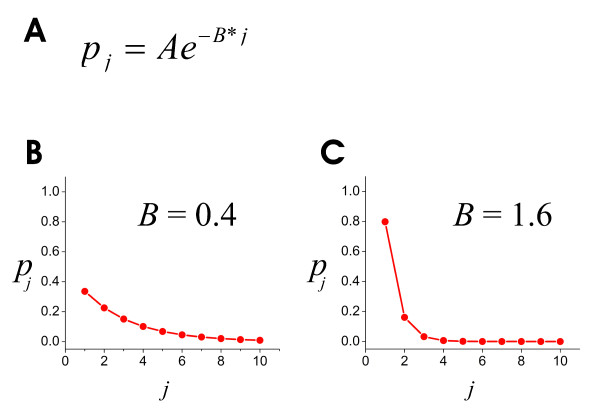
**The weighting function**. A, Equation used to generate weights in model. Here, "weight" refers to the probability that unit *i*, when active, will transmit to unit *j *in the next time step. B, Example of weighting function produced when *B *= 0.4. C, Example of weighting function produced when *B *= 1.6.

Using this model, we next explored how the distribution of connection weights affected the distribution of groups of significantly repeating avalanches. Connection weights in the model were given by a weighting function (see Methods) shown in figure 6. Note that the steepness of the curve was determined by the exponent *B*. We sought to determine which value of *B *produced a distribution of groups of repeating avalanches that best matched the data. To do this, the model was run ten times for each value of *B*, each time for a period that simulated one hour of real time (900,000 bins of 4 ms each), and all groups of statistically significant avalanches were extracted. We examined only groups containing avalanches of length 2 through 9. Avalanches of length 1 had no temporal extent, and were excluded because their occurrences did not clearly depend on the connection strengths between units. Statistically significant groups with avalanches longer than 9 frames were not observed in the data or in the simulations.

**Figure 7:**
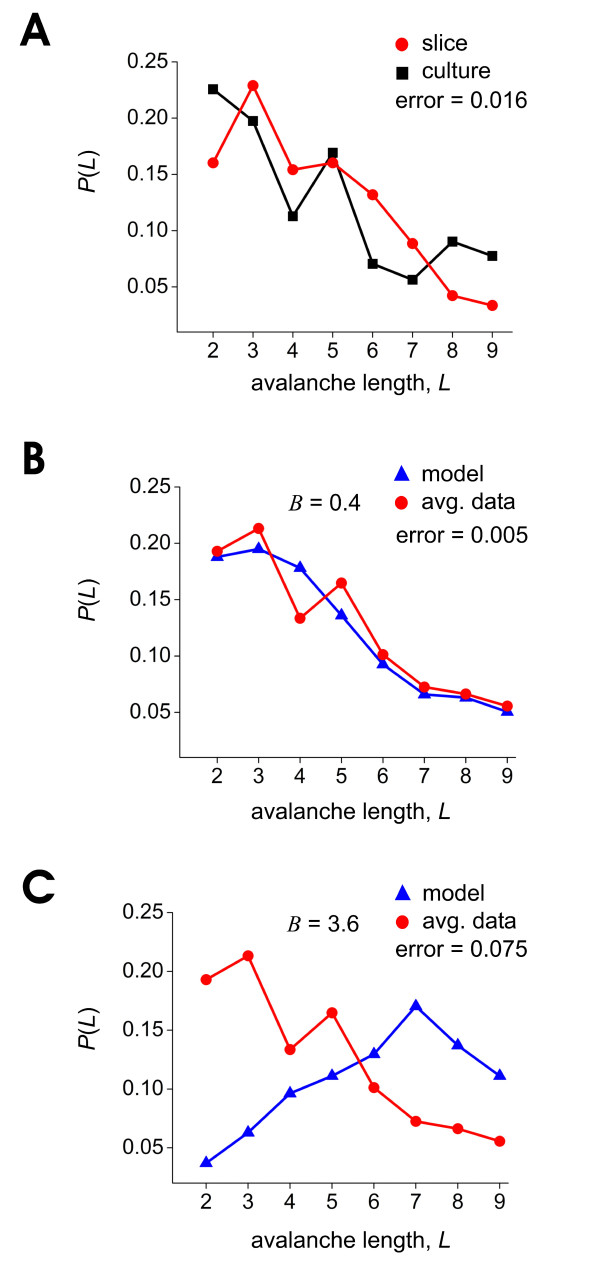
**Changes in the exponent *B *can drastically affect the distribution of significant avalanches**. A, Probability of observing a group of statistically significant avalanches of length *L*, plotted against *L*. Plot shows data produced by acute slices (n = 7) and organotypic cultures (n = 7; data reproduced from figure 10 of [17]). Note decline in probability of observing a group of significant avalanches with increasing *L*. Sum of squares difference between these two data distributions was 0.016, and is taken as an estimate of the intrinsic error of the data itself. B, Distribution of groups of significant avalanches produced by model with weighting function having exponent *B *= 0.4, plotted on top of average distribution from slice and culture data. Sum of squares error between data and model was 0.005. C, Same as in B, but here model weighting function had an exponent of *B *= 3.6. Sum of squares error was 0.075. Note that changes in the exponent *B *can produce large changes in the distribution of groups of significant avalanches.

Figure 7A shows that the number of groups of statistically significant avalanches produced by acute cortical slices (n = 7) and organotypic cortical cultures (n = 7; data from [17]) declines with the length, *L*, of the avalanches. The fact that both data sets show this general trend suggests that similar mechanisms underlie these avalanches in slices and cultures. The sum of squares error between these two data sets was 0.016, and was taken as an estimate of the general variability of the data.

**Figure 8:**
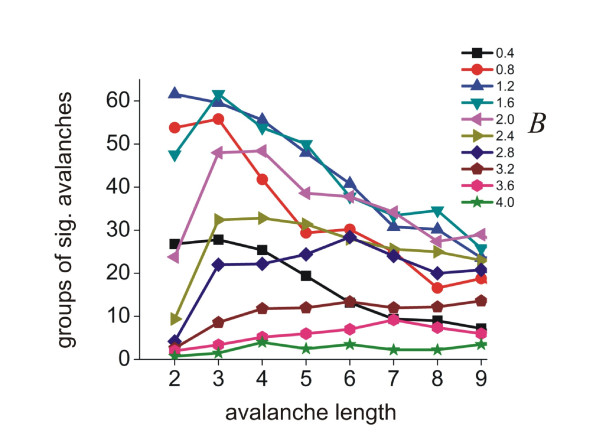
**Number of groups of avalanches**. Number of groups of significant avalanches for all values of the exponent *B*. Note wide variation in total number of groups and in shapes of curves.

The exponent of the weighting function had a profound effect on the distribution of groups of significant avalanches. Low values of *B *(e.g., 0.4) produced distributions of groups of avalanches that also declined with *L *(figure 7B). High values of *B *(e.g., 3.6) produced distributions with the opposite trend (figure 7C), contrary to what was seen in the data. Figure 8 shows the distributions produced by all values of *B*.

**Figure 9:**
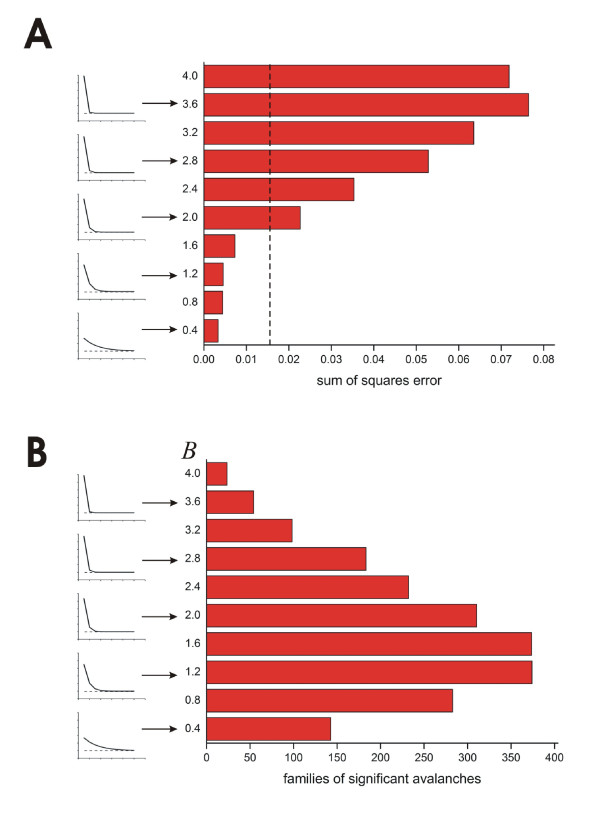
**The exponent *B *affects the fit between the model and the data, and the number of groups of significant avalanches produced by the model**. A, Left column shows weighting functions produced by different values of *B*. Right histogram shows sum of squares error between model and data distributions of groups of significant avalanches. Dashed line at 0.016 is the error between the data produced by acute cortical slices and the data produced by organotypic cultures. This value is taken as an estimate of the intrinsic error of the data itself. Weighting functions that cause the model to produce distributions of groups of significant avalanches with error less than this intrinsic error are considered to have a good fit (*B *≤ 1.6). B, Again, left column shows weighting functions produced by different values of *B*. Right histogram shows total number of groups of significant avalanches produced by models with different values of *B*.

The sum of squares error between the data and all model distributions is plotted in figure 9A. Low values of *B *(0.4 - 1.6) produced relatively good fits to the data that had errors less than the intrinsic error of the data itself. Values of *B *greater than 1.6 produced fits with errors that exceeded 0.016, the variability of the data itself. From this we conclude that only weighting functions with *B *in the range of 0.4-1.6 could reasonably fit the data.

### Effect of the exponent *B *on the number of groups

**Figure 10:**
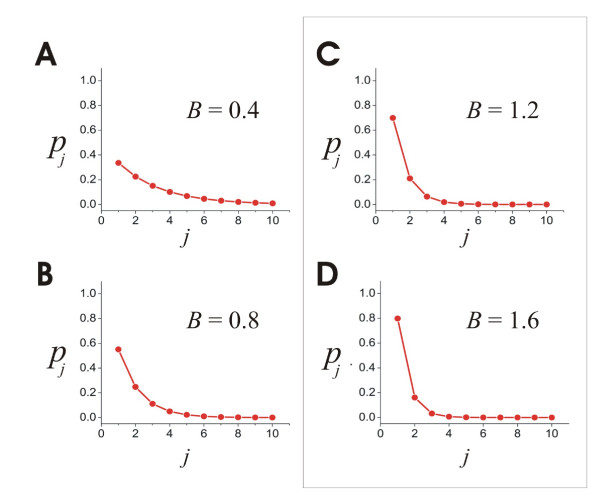
**Some weighting functions fit the data and maximize the number of repeating activity patterns**. A, B: Weighting functions with exponents *B *= 0.4 and *B *= 0.8 both produce error values lower than the error between slice and culture data, but do not produce the most groups of significant avalanches. C, D: Weighting functions with exponents *B *= 1.2 and *B *= 1.6 (outlined by box) have error values lower than the error between slice and culture data and produce the most groups of significant avalanches.

We also counted the total number of groups of statistically significant avalanches produced by the model for all values of *B*. Figure 9B shows the maximum number of groups of significantly repeating avalanches varied widely and peaked at *B *= 1.2 and *B *= 1.6. Figure 10 shows all the weighting functions that were considered good fits for the data. Interestingly, two of these functions were also found to produce the largest number of groups of significantly repeating avalanches. Thus, two of the weighting functions that were best-fitting were also optimal.

## Discussion

### Relation to other work

Our results are consistent with several experimental studies that have examined the distribution of connection strengths in living neural networks. Song and colleagues [14] used patch clamp recording in cortical slices to measure the amplitudes of post synaptic currents produced in layer V pyramidal neurons by neighboring neurons. They found that the distribution of amplitudes was approximately lognormal, with only a few strong connections. Brunel and colleagues, working in the cerebellum, reported similar findings [9]. They found that the distribution of synaptic strengths from granule cells onto Purkinje neurons also had very few strong synapses. Although these two studies examined synaptic connection strengths, their results are similar to those of Pajevic and Plenz [36], who reconstructed activity flow in cortical slice networks from field potential data. Pajevic and Plenz [36] found that connection strengths from a single electrode were distributed approximately exponentially, again with only a few strong connections.

Our results are also in agreement with modeling studies. Gardner [11] analytically derived the distribution of connection strengths that would maximize storage capacity in the perceptron, a type of feed-forward neural network model that can be used for pattern classification [37,38]. This distribution again had very few strong connections. Brunel and colleagues noted the correspondence between the optimal weight distribution for the perceptron and their findings in the cerebellum [9]. In the recurrent Hopfield neural network model [8], storage capacity is actually reduced if each unit has too many strong connections. This is because an excess of strong connections produces false associations between patterns that are actually distinct. When such an over connected network is annealed from a given starting configuration, it will often converge to a spurious hybrid state that is a mixture of two or more previously learned states. This problem of saturation in neural network models has been noted by several groups [39,40], whose results suggest that optimal storage is best achieved when strong connections are sparsely distributed. All of these findings are consistent with the results of the present study.

None of these previous reports, however, examined how the distribution of weights was related to spatio-temporal activity patterns. This is an important topic for exploration because in vivo studies have increasingly suggested that memories in behaving animals are linked to spatio-temporal activity patterns, and not to static configurations of active neurons [21,24-26]. Our work builds on previous studies by explicitly linking the capacity of a network model to store such spatio-temporal patterns to the weight distribution between units in the network. We also go beyond previous work by exploring how connection weights affect the distribution of groups of significantly repeating spatio-temporal patterns. We accomplish this by recording significantly repeating spatio-temporal activity patterns of all lengths over several hours, something that is presently difficult to do in vivo and has not yet been reported. In this respect, an in vitro network preparation is useful as it provides a stable platform from which to examine all patterns of activity produced by a network over a long time without interruption from outside perturbations.

### Validity and limitations

This work does have several limitations, though. First, the spatio-temporal activity patterns reported here were all recorded from isolated cortical networks. The activity in these networks was therefore not produced by sensory stimulation, and modulatory systems that are normally present in vivo were absent. In addition, the pattern of synaptic connections present in vivo is not necessarily reproduced in vitro. For neuronal cultures, connections are not formed in response to sensory experience. For acute slices, some axons and dendrites are likely to be cut. Second, the signals analyzed here were all LFPs, which are difficult to attribute to individual neurons or synapses. Caution should therefore be exercised when using the results of the present study to predict phenomena in vivo.

Despite these limitations, in vitro preparations are still widely used as models of local cortical networks because many of the features characteristic of neocortex are preserved: neuronal morphology [41], cytoarchitecture [42,43], gross intracortical connectivity [44], and intrinsic electrophysiological properties [45-47]. In addition, the reproducible activity patterns seen in cultures and in slices are similar to each other [15,17,48], and are remarkably similar to activity patterns seen in vivo [49-51].

With regard to LFPs, several recent reports indicate that bursts of action potentials, like the kind that could produce LFPs, as well as LFPs themselves, often contain more information about the state of a network than do single action potentials [52-55]. An analysis of LFPs in cortical slices and cultures is therefore expected to provide insights into information retention at the local network level. It is also interesting to note that the LFP bursts that we call neuronal avalanches can be induced in acute slices by the application of NMDA and dopamine agonists [28,56]. These agents also have been found to induce UP states in vivo [57], suggesting that avalanches may be related to UP states. Although this link was not explored in this paper, this potential connection further motivates the investigation of these LFP bursts.

One might also ask why we did not attempt to reconstruct weight distributions from the data. Although such a reconstruction is possible and recently has been done for cortical slice networks [36], it is important to note that reconstruction alone would not have allowed us to ask how changes in the weight distribution affect the storage capacity of the network. For our purposes, it was important to have a tunable network model where we could explore storage capacity as a function of a single variable. We adopted this approach because it is presently unfeasible to experimentally tune the weight distribution on all nodes in living neural networks. As it turns out, the number of connections we use in our model, as well as the shape of the weight distribution, are consistent with the results of reconstruction [36]. We also did not attempt to explicitly model the small-world degree distribution found by Pajevic and Plenz [36]. This would have involved introducing heterogeneity into our model, and would have complicated the interpretation of our results. Instead, we chose to treat all nodes as equivalent and gave them random connectivity. The degree distribution of a random network with a mean degree of 10 would be similar in shape to that found by reconstruction [36], suggesting that our results are unlikely to be affected much by this difference. Nevertheless, this is an interesting topic that could be explored more fully in future studies.

The model that we propose here is phenomenological and is not yet clearly linked to biophysical mechanisms. Some of our previous modeling work [58-60] has suggested ways in which biophysical mechanisms like firing rate homeostasis and conservation of synaptic strengths might play a role in tuning the network to the point where it would produce avalanches. These predictions are testable, indicating that the model eventually may be linked to mechanisms at scales smaller than the network.

### Implications

Multielectrode recordings from behaving animals indicate that reproducible spatio-temporal patterns of neural activity are used to represent specific items in memory [21,24-26]. These findings suggest that the significantly repeating avalanche patterns that we find in both our in vitro data and in our models may be used as a platform from which to explore information retention in local cortical networks. If neuronal avalanches can be used for this purpose, then our findings may place constraints on how learning could occur in local cortical networks. It is presently unclear how a network could maintain an optimal distribution of weights in the face of learning, if learning involves strengthening or weakening of existing connections. Learning could disrupt optimal distributions, pushing the network away from the point of maximum storage capacity. It is possible that a rescaling process could allow individual connections to change strength while approximately preserving the overall distribution near an optimal point. There is some support for rescaling of synaptic strengths in response to changes in overall activity levels [61], as well as support for conservation of synaptic strengths in the face of plasticity [62]. Modeling studies would be needed to determine whether such mechanisms could preserve an optimal distribution in the presence of synaptic plasticity [59].

It is also interesting to note that network studies in other areas of biology have found weight distributions that have relatively few strong connections [63,64], hinting that this distribution is perhaps of more general significance. Kauffman and colleagues have shown that two connections per node in random Boolean networks leads to systems that are poised at the edge of chaos, where adaptability is maximized without introducing instability [65]. This intriguing conjecture has yet to be explored in neural networks.

Finally, we would like to note that Hebb's theories on memory did not just emphasize a particular synaptic learning rule, but a process whereby groups of cells, or "assemblies" could activate each other in sequences [2]. The order in which these assemblies are activated is expected to have important consequences for memory. Both theoretical [66] and experimental [67,68] work has begun to probe this idea (see also [69]). Because modeling studies suggest that the distribution of connection strengths influences the dynamics of activation [70], it is likely that the distribution of strengths will also place constraints on the order in which assemblies can be activated.

## Conclusions

The main finding of this study is that the set of connection strengths that *best fits *the distribution of groups of significant avalanches also produces the *largest number *of groups of significant avalanches. This suggests that the set of connection strengths actually used in cortical slice networks is the one that maximizes information storage capacity. This set of connection strengths is skewed, with only a few strong connections and many weak ones. Fixed distributions impose constraints on learning, however. We conclude that the network must have mechanisms for preserving the overall weight distribution while allowing individual connection strengths to change with learning.

## Methods

### Data used in this study

The output of the model was compared to two data sets, one recorded from acute slices as a part of this study and one recorded from organotypic cultures as part of a previously published study [17]. We compared output from the model to data from two different preparations (acute slices and organotypic cultures) to test its generality. Note that we did not re-analyze the older data set, but merely compared the output from the model to previously published graphs.

### Tissue preparation and recording

All neural tissue was prepared according to guidelines from the National Institutes of Health and all animal procedures were approved by the Indiana University Animal Care and Use Committee. Acute slices were prepared as previously reported [31]. Sprague-Dawley rats 14-35 days old (Harlan) were deeply anesthetized with Halothane and then decapitated. Brains were removed and immediately placed for 3 mins in ice-cold artificial cerebrospinal fluid (ACSF) containing in mM: Sucrose 125, KCl 3, NaH_2_PO_4 _*H_2_O 1.25, NaHCO_3 _26, MgSO_4 _*7H_2_O 2, CaCl_2 _*2H_2_O 2, D-glucose 10, saturated with 95% O_2_/5%CO_2_. After cooling, brains were blocked into ~5 mm^3 ^sections containing somatosensory cortex, striatum and thalamus. Blocks were then sliced into coronal sections with a thickness of 250 μm using a tissue slicer (Vibratome). After cutting, slices incubated for ~1 hr at room temperature in ACSF with the same ingredients as listed above, but with 125 mM NaCl substituted for 125 mM sucrose to restore Na^+ ^and allow cells to fire action potentials again. After incubation, slices were adhered to microelectrode arrays with a solution of 0.1% polyethelinamine that had been previously applied and let to dry for 2 hrs [71]. We attempted to place the tissue so that neocortical layers I-V covered the array. Slices were maintained thermostatically at 37°C and were perfused at 1.0 ml/min with excitable ACSF solution containing 5 mM KCl and 0 mM Mg^+2 ^during recording, which typically lasted 5 hrs. These external ionic concentrations are known to produce robust local field potential (LFP) activity in cortical brain slices [72,73].

### Electrode arrays

Recordings were performed on microelectrode arrays purchased from Multichannel Systems (Reutlingen, Germany). Each array had 60 electrodes, and each electrode was 30 μm in diameter. For acute slice recordings, we used electrodes that came to a point 30 μm high, but in the previous organotypic culture recordings we used electrodes that were flat [28]. Electrodes were arranged in a square grid with 200 μm spacing between electrodes (figure 1A).

### Local field potential (LFP) detection

Extracellular activity from acute slices was recorded in the same manner as the previous organotypic culture recordings [17,28,31]. Activity was sampled from all 60 electrodes at 1 kHz and amplified before being stored to disk for offline analysis. Local field potentials (LFPs) that showed sharp negative peaks (figure 1B) below a threshold set at 3 standard deviations of the signal were marked, and the time of the maximum excursion was recorded as the time of that LFP (figure. 1C). Time points were binned at 4 ms resolution, as this was previously shown to match the average time between successive LFP events across electrodes [28].

### Characterizing multielectrode activity

In characterizing network activity, we closely followed the methods first described in [17,28]. The configuration of active electrodes during one time step is called a *frame*. An *avalanche *is a sequence of consecutively active frames that is preceded by a blank frame and terminated by a blank frame. The *length *of an avalanche is given by the total number of active frames and the *size *of an avalanche is given by the total number of electrodes activated during the avalanche. For example, the sequence of frames shown in figure 2 is an avalanche of length 5 and size 9. These definitions allowed us to compare the distribution of avalanche sizes generated by the model with those from the data. In particular, we wanted to be sure that the number of electrodes activated in the simulated avalanches was distributed according to a power law, as was seen in the data [27,28,33].

### Detecting groups of avalanches

We were also interested in whether the model could reproduce the significantly repeating avalanches that were generated by cortical tissue. We here describe our methods for identifying these significant avalanches. During spontaneous activity, avalanches of many different lengths were produced. We compared only avalanches of the same length, measuring their similarity with each other. To measure similarity, each 8 × 8 frame was "unfolded" into a 1 × 64 vector (the four corner electrodes were absent from the array, leaving the possibility of only 60 active electrodes). An avalanche of length *l *frames was then formed by concatenating *l *linear vectors of 1 × 64 into a single vector of 1 × 64 *l*. These vectors were then compared to each other for similarity using a Boolean measure, first used in this context by Jimbo and colleagues [74]. Boolean similarity *S *between two vectors *X *= (*x*_1_, *x*_2_, *x*_3_, ... *x*_*n*_) and *Y *= (*y*_1_, *y*_2_, *y*_3_, ... *y*_*n*_) ranged from 0 (least similar) to 1 (perfectly similar) and was given by their intersection divided by their union:(1)

where ⟨·,·⟩ indicates a dot product. Note that this number is sometimes called the Jacquard coefficient in other literature [75]. Once all avalanches of the same length were compared for similarity, they were assembled into a matrix (figure 4A). Ordering this matrix was accomplished by a greedy algorithm (the "dendrogram" function in Matlab) that placed avalanches with high similarity scores next to each other along the margins of the matrix. Other methods like simulated annealing took longer but produced only slightly better results [17]. Figure 4B shows an example of a matrix ordered with the dendrogram algorithm. Note the dark squares of various sizes along the diagonal of the matrix, indicating avalanches sharing high mutual similarity. Boxes could be drawn around these squares to separate them from the surrounding lighter areas of the matrix. In this way groups of mutually similar avalanches could be extracted. A contrast function found the grouping that maximized differences in total similarity between regions within boxes and regions outside of boxes. The contrast for a particular set of boxes was given by:(2)

where *D*_*in *_= (the sum of similarity values inside all boxes)/(the number of similarity values inside all boxes) and *D*_*out *_= (the sum of similarity values outside all boxes)/(the number of similarity values outside all boxes). Maximum contrast was obtained when there was a preponderance of dark pixels within the boxes and lighter pixels outside the boxes. The arrangement of boxes that maximized the contrast function was chosen as the best possible grouping. These groups of avalanches were next compared to groups produced by chance to assess statistical significance.

### Determining statistical significance

**Figure 11:**
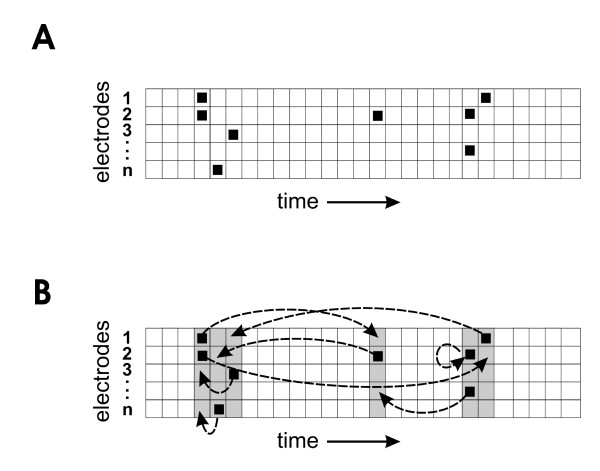
**Shuffling scheme**. A, Representation of unshuffled network activity in raster format. Black squares indicate LFP signals over threshold; each row is activity from an electrode; each column is one time bin. B, Shuffling of activity. Gray columns indicate time bins where at least one electrode was active in the unshuffled data. Dashed arrows indicate example shuffles allowed by this procedure. Activity from a given electrode can only be shuffled to another gray time bin in the same row.

To identify groups of avalanches that were statistically significant, we compared the similarity values from groups of avalanches from actual data to similarity values from groups of avalanches produced by random data sets. Because we desired to compare the output of the model to previously published data [17], we followed the most stringent method of shuffling adopted in our previous work (figure 11). In that scheme, activity on a given electrode could only be shuffled to another time bin on the same electrode, thus preserving the firing rate on each electrode. The time bins into which activity was shuffled were restricted to those bins that already had some activity on at least one other electrode, thus approximately preserving the overall temporal profile of network activity. When compared to frame shuffling, electrode shuffling or jittering, this method was found to be the least likely to produce false positives [17]. Once shuffled according to this method, 20 sets of randomized data were compared to the actual data. Groups of avalanches in the actual data that had a higher average similarity than all groups of avalanches of the same length found in the shuffled data were considered significant at the p < 0.05 level. Shuffled data sets served as controls to ensure that the effects we observed could not have been attributed to chance.

### Model of slice network activity

The previously published model [27] qualitatively captured both the power law distribution of avalanche sizes [28] and the groups of significantly repeating avalanches [17] seen experimentally. This model is formally equivalent to a critical branching process [35], which has found application in diverse areas of physics. Here we describe the model and our modifications to investigate the effects of the weight distribution. The model represented each of the 60 recording electrodes by a binary processing unit that could be either on (1) or off (0), since an electrode could receive either suprathreshold or subthreshold input. We approximated the electrode array with an 8 × 8 sheet of processing units where each unit was randomly connected to 10 other units, which gave the network a recurrent, rather than a feed-forward, architecture. Each connection from unit *i *to unit *j *had a probability *p*_*ij *_of transmitting that was randomly chosen from a weighting function (described below) and then fixed. The sum of probabilities emanating from a unit *i *determined the branching parameter of that unit:(3)

where 0 ≤ *p*_*ij *_≤ 1 and 0 ≤ *σ*_*i *_≤ 10. Pajevic and Plenz [36] reconstructed activity flow in cortical slice networks recorded with 60 electrode arrays. In their work, it was found that the distribution of connections per electrode peaked near a mean value of 10. Although we did not here attempt to reproduce the distribution of connections that they found, the number of connections per node in our model was set at 10, in approximate agreement with their findings. Note that *σ*_*i *_was equivalent to the expected number of descendants an active unit *i *produced. When *σ *<1, the network was subcritical and activity died out; when *σ *= 1, the network was critical and activity was approximately sustained; when *σ *>1, the network was supercritical and activity increased. In all of the simulations described here, we set the model to operate in the critical regime (*σ *= 1), as that provided the best fit to the avalanche size distribution observed in the data [27,33]. We adopted this approach because recent work has shown that it is difficult to estimate *σ *correctly from firing times in the data without knowing details about the geometry of the network [76]. Each unit had a small probability of becoming spontaneously active at any time step, given by *p*_*spont*_. We chose *p*_*spont *_= 0.005 to reproduce the average firing rates observed in the data, and found that our results did not depend strongly on the choice of this parameter. Units could also become active through driven activity. For example, unit *j *at time step *t *+ 1 became active if unit *i *in the previous time step *t *was active and the connection between them transmitted. A connection transmitted if *rand *≤ *p*_*ij*_, where *rand *was a uniformly distributed random number drawn from the interval [0, 1]. Processing units updated at each time step to simulate the propagation of activity through the network. After becoming active, a unit would become inactive, or refractory, for the next 5 time steps. This refractory period was chosen to mimic that found in the data. Even though the network was stochastic, certain preferred avalanches of activity could develop as a result of the fixed underlying transmission probabilities. This led to significantly repeating avalanches mentioned earlier.

### The weighting function

In previous models [27,33], the distribution of connection strengths was chosen from a uniform random distribution; here we investigated how the choice of this distribution affected model performance. As many studies report weight distributions with a nearly exponential form [9,11,14,77,78], we adopted a weighting function where transmission probabilities *p*_*ij *_were determined by the following:(4)

The denominator was just a normalizing term to make the sum of the *p *values equal one. When *B *= 0, the distribution was homogeneous; as *B *was increased greater than zero, the distribution of *p *values became increasingly skewed. We changed *B *to see how it affected avalanches produced by the network model. In this manner, we were able to identify the values of *B *that best fit the data and that produced the largest number of groups of significantly repeating avalanches.

### Running simulations

Each simulation was run for 900,000 time steps so as to model 1 hr of data binned at 4 ms. We examined ten different values of *B*, and for each value we ran 10 simulations. All software was written in Matlab and run on PCs.

## Authors' contributions

WC, JH, AT and JMB conceived the experiments. WC, JH and AT performed the experiments. WC and JMB performed the simulations and wrote the manuscript. WC, JH, and AT read the manuscript and provided critical comments. All authors approved the final manuscript.
